# Interactive Effects of Racial Identity and Repetitive Head Impacts on Cognitive Function, Structural MRI-Derived Volumetric Measures, and Cerebrospinal Fluid Tau and Aβ

**DOI:** 10.3389/fnhum.2019.00440

**Published:** 2019-12-20

**Authors:** Michael L. Alosco, Yorghos Tripodis, Inga K. Koerte, Jonathan D. Jackson, Alicia S. Chua, Megan Mariani, Olivia Haller, Éimear M. Foley, Brett M. Martin, Joseph Palmisano, Bhupinder Singh, Katie Green, Christian Lepage, Marc Muehlmann, Nikos Makris, Robert C. Cantu, Alexander P. Lin, Michael Coleman, Ofer Pasternak, Jesse Mez, Sylvain Bouix, Martha E. Shenton, Robert A. Stern

**Affiliations:** ^1^Boston University Alzheimer’s Disease Center and Boston University CTE Center, Boston University School of Medicine, Boston, MA, United States; ^2^Department of Neurology, Boston University School of Medicine, Boston, MA, United States; ^3^Department of Biostatistics, Boston University School of Public Health, Boston, MA, United States; ^4^Psychiatry Neuroimaging Laboratory, Department of Psychiatry, Brigham and Women’s Hospital, Harvard Medical School, Boston, MA, United States; ^5^cBRAIN, Department of Child and Adolescent Psychiatry, Psychosomatic, and Psychotherapy, Ludwig-Maximilian-University, Munich, Germany; ^6^CARE Research Center, Massachusetts General Hospital, Boston, MA, United States; ^7^Biostatistics and Epidemiology Data Analytics Center, Boston University School of Public Health, Boston, MA, United States; ^8^Department of Radiology, University Hospital, LMU Munich, Munich, Germany; ^9^Department of Radiology, Brigham and Women’s Hospital, Harvard Medical School, Boston, MA, United States; ^10^Center for Morphometric Analysis, Massachusetts General Hospital, Boston, MA, United States; ^11^Center for Neural Systems Investigations, Massachusetts General Hospital, Boston, MA, United States; ^12^Concussion Legacy Foundation, Boston, MA, United States; ^13^Department of Neurosurgery, Boston University School of Medicine, Boston, MA, United States; ^14^Department of Neurosurgery, Emerson Hospital, Concord, MA, United States; ^15^Department of Radiology, Center for Clinical Spectroscopy, Brigham and Women’s Hospital, Harvard Medical School, Boston, MA, United States; ^16^VA Boston Healthcare System, U.S. Department of Veteran Affairs, Brockton, MA, United States; ^17^Department of Anatomy and Neurobiology, Boston University School of Medicine, Boston, MA, United States

**Keywords:** American football, biomarkers, chronic traumatic encephalopathy, cognitive function, concussion, magnetic resonance imaging, race, subconcussion

## Abstract

**Background:**

Factors of increased prevalence among individuals with Black racial identity (e.g., cardiovascular disease, CVD) may influence the association between exposure to repetitive head impacts (RHI) from American football and later-life neurological outcomes. Here, we tested the interaction between racial identity and RHI on neurobehavioral outcomes, brain volumetric measures, and cerebrospinal fluid (CSF) total tau (t-tau), phosphorylated tau (p-tau_181_), and Aβ_1__–__42_ in symptomatic former National Football League (NFL) players.

**Methods:**

68 symptomatic male former NFL players (ages 40–69; *n* = 27 Black, *n* = 41 White) underwent neuropsychological testing, structural MRI, and lumbar puncture. FreeSurfer derived estimated intracranial volume (eICV), gray matter volume (GMV), white matter volume (WMV), subcortical GMV, hippocampal volume, and white matter (WM) hypointensities. Multivariate generalized linear models examined the main effects of racial identity and its interaction with a cumulative head impact index (CHII) on all outcomes. Age, years of education, Wide Range Achievement Test, Fourth Edition (WRAT-4) scores, CVD risk factors, and *APOE*ε4 were included as covariates; eICV was included for MRI models. *P*-values were false discovery rate adjusted.

**Results:**

Compared to White former NFL players, Black participants were 4 years younger (*p* = 0.04), had lower WRAT-4 scores (mean difference = 8.03, *p* = 0.002), and a higher BMI (mean difference = 3.09, *p* = 0.01) and systolic blood pressure (mean difference = 8.15, *p* = 0.03). With regards to group differences on the basis of racial identity, compared to White former NFL players, Black participants had lower GMV (mean adjusted difference = 45649.00, *p* = 0.001), lower right hippocampal volume (mean adjusted difference = 271.96, *p* = 0.02), and higher p-tau_181_/t-tau ratio (mean adjusted difference = −0.25, *p* = 0.01). There was not a statistically significant association between the CHII with GMV, right hippocampal volume, or p-tau_181_/t-tau ratio. However, there was a statistically significant Race x CHII interaction for GMV (*b* = 2206.29, *p* = 0.001), right hippocampal volume (*b* = 12.07, *p* = 0.04), and p-tau_181_/t-tau ratio concentrations (*b* = −0.01, *p* = 0.004).

**Conclusion:**

Continued research on racial neurological disparities could provide insight into risk factors for long-term neurological disorders associated with American football play.

## Introduction

Exposure to repetitive head impacts (RHI) from contact sports has been associated with later-life neurological disorders, including chronic traumatic encephalopathy (CTE) and other neurodegenerative diseases (McKee et al., 2013, 2016; Bieniek et al., 2015; Ling et al., 2017; Mez et al., 2017b; Adams et al., 2018; Tagge et al., 2018; Alosco et al., 2019a; Stern et al., 2019). Autopsy studies of convenience samples suggest that professional American football players may be at high-risk for later-life neurological disorders (McKee et al., 2013; Mez et al., 2017b). *In vivo* studies show that former National Football League (NFL) players have worse cognition, as well as greater structural, functional, and molecular brain alterations (Didehbani et al., 2013; Hart et al., 2013; Strain et al., 2015; Koerte et al., 2016a; Alosco et al., 2018c, 2019b; Lepage et al., 2018). These effects might extend to high school and college football players (Mez et al., 2017b), and other contact sport athletes (Koerte et al., 2012, 2015, 2016b; Ling et al., 2017). Yet, not all individuals exposed to RHI develop neurological disorders (Casson et al., 2014; Solomon et al., 2016; Deshpande et al., 2017; Baker et al., 2018; Willer et al., 2018; Zivadinov et al., 2018). Among those that do, there is heterogeneity in disease presentation, suggesting other risk factors are at play (McKee et al., 2013; Stern et al., 2013; Alosco et al., 2017c, 2018b; Mez et al., 2017b). These may include: age (McKee et al., 2013; Alosco et al., 2018c), age of first exposure (AFE) to RHI (Stamm et al., 2015a, b; Alosco et al., 2017b, 2018b; Schultz et al., 2017), cognitive reserve (Alosco et al., 2017c), and genetics (Stern et al., 2013; Cherry et al., 2018).

The effect of exposure to RHI on neurological disorders may also be modified by factors associated with race. Note that because the term *African-American* historically refers primarily to individuals descended from enslaved Africans in North America, we will use the term *Black* as it is inclusive of all individuals who are descended from sub-Saharan Africa regardless of specific ancestry or position within the broader African diaspora. Individuals who identify as Black are at increased risk for cognitive impairment, cognitive decline, and dementia, including Alzheimer’s disease (AD) dementia (Potter et al., 2009; Barnes and Bennett, 2014; Gross et al., 2015; Hohman et al., 2016; Zahodne et al., 2016). *In vivo* MRI studies in older adults show that Black identifying individuals exhibit increased volume of white matter hyperintensities (Brickman et al., 2008) and differences in brain volumes compared to Whites (Sencakova et al., 2001; Brickman et al., 2008; Waldstein et al., 2017; Morris et al., 2019). Autopsy studies also link Black racial identity with increased risk for neuropathologic changes of AD and related diseases, including cerebrovascular disease (Graff-Radford et al., 2016; Filshtein et al., 2019). Although, some studies have failed to find an association between Black racial identity and AD pathologic changes (Riudavets et al., 2006; Wilkins et al., 2006; Morris et al., 2018) and risk for dementia (Fillenbaum et al., 1998). A recent study also reported *decreased* CSF t-tau and p-tau_181_ in Blacks compared to Whites (Morris et al., 2019). These racial neurological disparities have been attributed to the association of Black racial identity with lifestyle behaviors across the life course known to influence cognitive aging (e.g., poor diet, sedentary behaviors) (Morris et al., 2018), cardiovascular disease (CVD) (Newman et al., 2005; Jackson et al., 2013; Benjamin et al., 2019), genetics (e.g., *APOE*ε4 or *ABCA7* status) (Reitz et al., 2013; Graff-Radford et al., 2016; Rajan et al., 2019), socioeconomic status (Zahodne et al., 2017), and other factors (Barnes and Bennett, 2014; Zahodne et al., 2017; Brewster et al., 2018). Many of these factors associated with racial identity may influence both clinical and neuropathological outcomes, whereas others (e.g., socioeconomic status) might have more specific impact on clinical function (e.g., neuropsychological test scores, dementia risk).

Previous studies report that Black participants have worse functional outcomes after acute traumatic brain injury (TBI) (Gary et al., 2009). Associations between Black racial identity and neurological outcomes have been examined in the setting of *active*, but not former, contact sport play. For example, among 403 active University of Florida student-athletes, individuals who identified as being Black demonstrated worse memory and speed performance on the Immediate Post-Concussion Assessment and Cognitive Testing (ImPACT) (Houck et al., 2018). In the subset of football players, Black racial identity only predicted worse processing speed. Research among active collision sport athletes also provides evidence for racial identity as an independent correlate of serum concentrations of S100B, UCH-L1, and Aβ and cognitive test scores (Asken et al., 2018b).

Racial differences have also been reported in aging former NFL player samples, including higher overall and CVD-related mortality rates in non-White former NFL players (Lincoln et al., 2018). In two recent studies of former NFL players from the NIH-funded Diagnosing and Evaluating Traumatic Encephalopathy using Clinical Tests (DETECT) study, our team found racial differences (via its inclusion as a model covariate) on white matter alterations on MRI (Alosco et al., 2018a), as well as on cerebrospinal fluid (CSF) levels of t-tau, p-tau_181_/t-tau, and sTREM2 (a marker of microglial activation) (Alosco et al., 2018c). These findings could have key implications given Blacks have been overrepresented (relative to the general population) in American football, particularly at elite levels, for the past 4–5 decades (Lehman et al., 2012; Lehman et al., 2016; Lapchick and Marfatia, 2017; Lincoln et al., 2018; NCAA, 2018). The contribution of racial identity to later-life neurological outcomes associated with exposure to RHI is unknown. Here, we tested the effects of the interaction between racial identity and a novel metric of exposure to RHI (the cumulative head impact index [CHII]) (Montenigro et al., 2017) on neurobehavioral outcomes, white and gray matter volume, and CSF concentrations of t-tau, p-tau_181_ and Aβ_1__–__42_ among symptomatic former NFL players from the DETECT study. Our overall hypothesis was that Black racial identity and the CHII would interact to have synergistic, negative impacts on neurobehavioral and neurological functioning.

## Materials and Methods

### Participants and Study Design

The sample included symptomatic former NFL players from the NIH-funded DETECT study. The primary objective of the DETECT study was to provide an initial examination of risk factors and *in vivo* biomarkers for CTE. Investigation of racial disparities was not a primary objective of the DETECT study and the current study represents secondary data analyses on differences between racial identity groups using data collected from DETECT. Recruitment and enrollment began in 2011 and concluded in 2015. Former NFL players were recruited using several different methods, including emails to and presentations for members of the NFL Players Association and/or NFL Alumni Association, social media postings through the Boston University (BU) Alzheimer’s Disease and CTE Centers, and word of mouth. Inclusion criteria for the former NFL players included: male, ages 40–69, a minimum of two seasons in the NFL and a minimum of 12 years of organized football, and self-reported complaints of cognitive, behavior, and/or mood symptoms at the time of telephone screening. For self-reported complaints, participants were eligible if they affirmatively responded to having any (i.e., absence or presence) cognitive, behavioral, or mood symptoms. Former NFL players were required to have subjective complaints in order to maximize the likelihood of having underlying CTE neuropathology to thereby facilitate risk factor and biomarker detection. Exclusion criteria included MRI and/or lumbar puncture (LP) contraindications, presence of another central nervous system (CNS) disease, and/or a primary language other than English. Twenty-eight same-age asymptomatic individuals without a history of contact sport participation or TBI were recruited to serve as a “control” group for the DETECT study (Stern et al., 2016; Alosco et al., 2017a, 2018a,c). However, this “control” group was not included in this study because only 1 participant self-identified as Black, thereby limiting the ability to conduct reliable study group comparisons as a function of racial identity.

All participants completed a single 2–3 days study visit, which involved administration of neuropsychological tests, neurological and psychiatric evaluations, LP, blood draw for *APOE* genotyping and other biomarker analysis, MRI, and history interview. Additional descriptions of the DETECT study have been reported previously (Alosco et al., 2017a, d, 2018a,c; Stern et al., 2016). All study protocols were approved by the Boston University Medical Center Institutional Review Board. The Partners Healthcare Human Research Committee approved all neuroimaging procedures. Participants provided written informed consent prior to participation.

### Measures

#### Neuropsychological and Neuropsychiatric Function

A neuropsychological test battery, and semi-structured interviews and self-report measures of neuropsychiatric function were administered to participants on a separate day from the LP. A full list of the tests administered as part of DETECT has been presented elsewhere (Alosco et al., 2017a). As part of this battery, participants were administered the Wide Range Achievement Test, Fourth Edition (WRAT-4). Standard scores of this measure were included in this study to operationalize early-life educational quality. The neuropsychological battery also included measures of attention, psychomotor speed, executive function, verbal and visual memory, language and visuospatial abilities, as well as gross motor functioning. Neuropsychiatric status was also evaluated, including symptoms of depression, suicidality, hopelessness, apathy, aggression, impulsivity, and hostility. To limit the number of analyses, principal component analysis (PCA) was performed to generate the following four neurobehavioral factor scores: psychomotor speed and executive function, verbal episodic memory, visual episodic memory, and behavioral/mood. The four factor scores were examined as outcomes. The derivation of the factor scores has been published elsewhere (Alosco et al., 2017a). Note that raw neuropsychological test scores were converted to standardized scores using normative data that accounted for age; for three of the tests, the normative data also accounted for education, along with age (i.e., Trail Making Test Parts A and B, and Controlled Oral Word Association Test). Race was not accounted for in the standardization procedures. The standardized scores were used in the PCA to form the factor scores.

#### Magnetic Resonance Imaging

All participants underwent structural MRI on a 3-Tesla MRI Scanner (Verio, Siemens Healthcare) with a 32-channel head array and the Syngo MR-B17 software suite. T1-weighted images were acquired with a magnetization-prepared rapid gradient echo sequence: TR = 1800 ms, TE = 3.36 ms, voxel size = 1 × 1 × 1 mm, acquisition matrix = 256 × 256, flip angle = 7°. The quality of the T1-weighted images was evaluated through visual inspection. Automated segmentation of volumes from the T1-weighted images was done using FreeSurfer 5.3^1^. This segmentation resulted in an automated Talairach transformation, segmentation of deep gray matter structures (including hippocampus), and parcellation of the cerebral cortex, based on gyral and sulcal structures (Desikan et al., 2006; Fischl et al., 2004a, b). Following the automated volumetric segmentation, quality assessment was performed to ensure the fit and completeness of the obtained FreeSurfer parcellation. Using this automated method in FreeSurfer, we obtained estimated total intracranial volume (eICV), total gray matter volume (GMV), total cortical white matter volume (WMV), total subcortical gray matter volume (sGMV), right and left hippocampal volume, and volume of white matter hypointensities. Manual correction of the hippocampus using Slicer 4.1^2^ (Fedorov et al., 2012) was performed using procedures described elsewhere (Lepage et al., 2018). eICV served as a covariate, whereas the other volumetric measures were examined as outcomes. The hippocampus and WM hypointensities were selected *a priori* due to previous work showing their associations with race (Brickman et al., 2008; Sencakova et al., 2001; Waldstein et al., 2017; Morris et al., 2019) and neurodegenerative disease (Bobinski et al., 2000; Sencakova et al., 2001; Tosto et al., 2015).

#### CSF Analytes

CSF (15–20 ml) was obtained by LP in the morning after overnight fasting. LPs were performed by the study neurologist using an atraumatic 25-guage Sprotte needle at either L3/L4 or L4/L5. After aspiration, approximately 10 ml of CSF was deposited into a polypropylene transfer tube and frozen at -80°C. Aliquots were shipped to the University of Pennsylvania for batch analysis of t-tau, p-tau_181_, and Aβ_1__–__42_. Methods of CSF biomarker analysis of t-tau, p-tau_181_, and Aβ_1__–__42_ are described elsewhere (Shaw et al., 2009; Grossman et al., 2014; Deters et al., 2017; Alosco et al., 2018c). Concentrations of these analytes served as outcomes for markers of neurodegeneration (t-tau), hyperphosphorylated tau (p-tau_181_), and amyloidosis (Aβ_1__–__42_). We also examined the p-tau/t-tau and p-tau/Aβ_1__–__42_ ratios. The p-tau/t-tau ratio has been shown to be sensitive to the detection of certain neurodegenerative diseases, such as frontotemporal lobar degeneration (FTLD) (Meeter et al., 2018). The p-tau/Aβ_1__–__42_ ratio has also been identified as a sensitive predictor of AD (Fagan et al., 2007; Racine et al., 2016). Inclusion of these ratios will thereby increase our ability to draw inferences on suspected underlying etiologies.

#### Cumulative Head Impact Index (CHII)

The CHII was used to quantify and define exposure to RHI, with higher CHII scores reflecting greater exposure to RHI (Montenigro et al., 2017). This index is based on the reported number of football seasons played, position[s] played, and levels played (e.g., youth, high school, college), as well as estimated head impact frequencies derived from published helmet accelerometer studies. For a description of the development of the CHII, refer to Montenigro et al. (2017). Because published helmet accelerometer data does not exist at the professional level, college-level estimates of head impact frequencies were extrapolated and applied to the current sample of former NFL players to estimate their post-college head impact frequencies.

#### Cardiovascular Disease Assessments

Blood pressure was taken for all participants and height and weight were measured to calculate BMI using the standard formula: weight (kg)/height^2^ (m). Diagnostic history of CVD risk factors were self-reported (absence or presence).

#### Racial Identity

Participants self-reported racial identity. They were asked, “What race do you consider yourself (primarily)?” Participants selected from the following options, consistent with racial categories from the 2010 US Census: White, Black or African American, American Indian, Alaska Native, Asian, Native Hawaiian/Pacific Islander, or Other. As with the 2010 Census, participants were able to select multiple racial identities, although this was not explicitly stated as an option. No other racial categories were selected by participants within the sample, and no individuals self-identified as both White and Black.

### Sample Size

The original sample included 96 symptomatic former NFL players from the NIH-funded DETECT study. The sample size for the present study was reduced to 68 (27 identified as Black and 41 as White) following restriction of the sample to those who had complete data on the primary independent and dependent outcomes of interest. Missingness was most common for the dependent variables. For the neurobehavioral factor scores, the sample size was reduced due to missing data on the individual tests that comprise the factor scores. There was missingness for MRI outcomes due to the exclusion of participants who did not undergo MRI (e.g., because of claustrophobia) and for those whose T1-weighted MRI acquisition was of inadequate quality due to motion artifact. There was missingness across the CSF analytes due to exclusion of participants who, following enrollment, refused to undergo an LP, as well as immunoassay quality control failure. There were no differences between the analytic sample (*N* = 68) and those excluded in terms of age, years of education, racial identity, or CHII score (*p*’s > 0.05 for all).

### Statistical Analyses

Independent sample t-tests and chi-square analyses were used to compare Blacks and Whites on demographic, athletic, medical, and *APOE* variables. Our previous research reported on the main effects of exposure to RHI on neurological outcomes in the DETECT sample (Alosco et al., 2018a, c). In this more focused sample of DETECT participants, we focused on the interaction between racial identity and the CHII. We conducted three separate multivariate generalized linear models (GLMs) with an unstructured outcome correlation matrix to determine the Race x CHII interaction effect on the following: (1) the four neurobehavioral factor scores (psychomotor speed and executive function, verbal episodic memory, visual episodic memory, and behavioral/mood); (2) MRI-derived volumetric measures (GMV, sGMV, WMV, right and left hippocampal volume, and WM hypointensities); and (3) CSF analytes (t-tau, p-tau_181_, p-tau_181_/t-tau, Aβ_1__–__42_, p-tau/Aβ_1__–__42_). The primary independent variables included the binary racial identity variable and the continuous CHII variable. GLMs were used because they do not make assumptions of the correlation structures of the outcomes and account for correlations between outcomes from the same participant. The models estimate all pairwise correlations between each predictor and each outcome. The outcomes were grouped in a multivariate model based on the construct being assessed. For example, for the neurobehavioral factor scores model, all four factor scores were included as dependent variables and we obtained an estimate for each predictor (e.g., each covariate, racial identity, Race x CHII) on each of the individual factor scores. Given the large number of hypotheses tested, we controlled the False Discovery Rate (FDR) using the BH procedure for all main and interactive associations of racial identity and Race x CHII (Benjamini and Hochberg, 1995).

All models controlled for age, years of education, WRAT-4 scores, *APOE* status (ε4 carriers vs. non-carriers), and CVD risk factors (i.e., BMI, systolic blood pressure, diagnostic history of diabetes). These covariates were *a priori* selected based on the literature showing their association with race and the neurological outcomes being studied. Estimated ICV was included as covariate for all models with MRI-derived volumetric measures as outcomes to account for individual differences in head size. As mentioned previously, the goal of the DETECT study was not to assess racial disparities and therefore detailed assessment of cultural, psychosocial, and socioeconomic variables was not performed.

## Results

### Sample Characteristics

Table 1 shows demographic, athletic, medical, and *APOE* characteristics for the Black and White former NFL players. Compared to White former NFL players, Black former NFL players were ∼4 years younger, had lower WRAT-4 scores, had a higher BMI, and had a higher systolic blood pressure. Note that the distribution of racial identity by within sample CHII severity scores (i.e., low, medium, high) included: Low had 9 White participants and 8 Black participants, medium had 17 White participants, and 15 Black participants, and the high exposure group had 15 White participants but only 4 Black participants. This is somewhat consistent with our Table 1 finding of lower, but not statistically significant different, CHII scores in Black participants compared to White participants. A descriptive summary of neurobehavioral functioning, MRI-derived volumetric measures, and CSF analyte concentrations for Black and White former NFL players is presented in Tables 2, 3.

**TABLE 1:** Sample characteristics.

	**Total sample**	**Black former**	**White former**	**P^a^**
	**(*N* = 68)**	**NFL players (*n* = 27)**	**NFL players (*n* = 41)**	
**Demographic and athletic history**				
Age, mean (SD) years	54.69 (8.16)	52.15 (7.63)	56.37 (8.15)	0.04
Education, mean (SD) years	16.50 (1.00)	16.44 (0.93)	16.54 (1.05)	0.71
Learning disability, n (%)	3 (4.6)	1 (4.0)	2 (4.9)	1.00
WRAT-4 standard scores, mean (SD)	98.91 (10.51)	94.07 (10.52)	102.10 (9.31)	0.002
Years of football play, mean (SD) years	18.54 (3.26)	18.37 (2.86)	18.65 (3.53)	0.74
Years in the NFL, mean (SD)	8.16 (2.61)	8.30 (2.69)	8.07 (2.58)	0.73
Cumulative Head Impact Index, mean (SD)	20394.65 (6500.78)	18798.24 (5843.83)	21445.95 (6762.75)	0.10
Age of first exposure to football, mean (SD)	11.71 (2.54)	12.15 (2.52)	11.41 (2.54)	0.25
Primary Position Group, n (%)				
Offensive line	20 (29.4)	6 (22.2)	14 (34.1)	0.25
Running back	7 (10.3)	8 (18.5)	2 (4.9)	
Tight end	4 (5.9)	1 (3.7)	3 (7.3)	
Offensive skill	1 (1.5)	0	1 (2.4)	
Defensive line	11 (16.2)	4 (14.8)	7 (17.1)	
Linebacker	13 (19.1)	4 (14.8)	9 (22.0)	
Defensive Back	12 (17.6)	7 (25.9)	5 (12.2)	
**Cardiovascular and *APOE* status**				
Body mass index, mean (SD) kg/m^2^	32.45 (4.86)	34.31 (5.47)	31.22 (4.02)	0.01
Systolic blood pressure, mean (SD)	130.53 (15.70)	135.67 (18.38)	127.15 (12.81)	0.03
History of hypertension, n (%)	31 (45.6)	13 (48.1)	18 (43.9)	0.69
History of diabetes, n (%)	5 (7.4)	4 (14.8)	1 (2.4)	0.08
*APOE* allele status, n (%) ε4 + (at least one copy)	22 (32.4)	11 (40.7)	11 (26.8)	0.23

**^*a*^Independent samples t-tests compared Black and White participants on all continuous outcomes. Chi-square was used to compare Black and White participants on primary position (linemen vs. other), history of hypertension, history of hypercholesterolemia, and APOE status (ε4 carriers vs. non-carriers). Fisher’s Exact Test (due to small cell sizes) was used to compare Black and White participants on history of learning disability, and history of diabetes. Final sample sizes after exclusion for missing data: Learning disability: N = 65.**

**TABLE 2:** Neuropsychological and structural MRI descriptives.

	**Black former NFL players**			**White former NFL players**		
	**Mean (*SD*)**	**95% CI**	**Median**	**Mean (*SD*)**	**95% CI**	**Median**
**Principal component factor z-scores^a^**						
Psychomotor speed/executive function	−0.47(0.78)	−0.78, −0.16	–0.56	0.29 (0.72)	0.06, 0.52	0.40
Verbal episodic memory	−0.24(0.58)	−0.47, −0.01	–0.38	0.03 (0.99)	−0.28, 0.34	–0.14
Visual episodic memory	0.13 (0.91)	−0.23, 0.49	0.24	0.06 (0.94)	−0.23, 0.36	0.18
Behavior/mood	0.25 (0.87)	−0.10, 0.59	0.19	0.32 (0.90)	0.04, 0.60	0.26
**Volumetric measures,^b^ mm^3^**						
Total gray matter volume	589163.82 (30457.77)	577115.12, 601212.51	588030.59	640877.24 (46013.98)	626353.44, 655401.05	634792.25
Total cortical white matter volume	464470.34 (41742.96)	447957.38, 480983.31	452985.42	496324.79 (45649.58)	481916.01, 510733.58	495873.36
Total subcortical gray matter volume	55937.15 (4584.69)	54123.51, 57750.79	55260.00	55448.95 (4001.86)	54185.95, 56712.09	55059.00
Right hippocampal volume	3312.04 (292.15)	3196.47, 3427.61	3309.00	3407.27 (420.40)	3274.57, 3539.96	3443.00
Left hippocampal volume	3333.89 (335.40)	3201.21, 3466.57	3379.00	3379.46 (392.35)	3255.62, 3503.30	3347.00
Volume of white matter hypointensities	2299.99 (2558.85)	1287.74, 3312.24	1470.90	2016.88 (1420.44)	1568.54, 2465.23	1622.50

**^*a*^Neuropsychological tests evaluated attention, executive function, verbal and visual episodic memory, language, and visuospatial function. Semi-structured interviews and self-report measures of neuropsychiatric function (e.g., depression, apathy, aggression) were completed. Raw scores were transformed to standard scores using normative data calibrated for age; for three tests (Trail Making Test Parts A and B, Controlled Oral Word Association Test) normative data accounted for education, along with age. Principal component analysis resulted in composite scores for psychomotor speed/executive function, verbal memory, visual memory, and behavior/mood domains. Lower scores represent worse performance for all factor scores, except for behavior/mood where higher scores reflect greater behavior and mood symptoms. *^*b*^Volumetric measures were derived from FreeSurfer. Manual correction of the hippocampus using Slicer 4.1 (http://www.slicer.org/) was performed.***

**TABLE 3:** Cerebrospinal fluid biomarker concentrations.

	**Black former NFL players**			**White former NFL players**		
	**Mean (*SD*)**	**95% CI**	**Median**	**Mean (*SD*)**	**95% CI**	**Median**
t-tau (pg/ml)	27.59 (7.26)	24.72, 30.46	27.00	37.24 (15.12)	32.47, 42.02	33.00
p-tau_181_ (pg/ml)	19.78 (7.59)	16.77, 22.78	21.00	18.46 (11.06)	14.97, 21.96	16.00
Aβ_1__–__42_ (pg/ml)	334.52 (72.16)	305.97, 363.06	333.00	376.49 (77.32)	352.08, 400.89	384.00
p-tau_181_/t-tau (pg/ml)	0.72 (0.25)	0.62, 0.82	0.74	0.51 (0.23)	0.43, 0.58	0.45
p-tau_181_/Aβ_1__–__42_ (pg/ml)	0.06 (0.02)	0.05, 0.07	0.06	0.05 (0.03)	0.04, 0.06	0.04

**t-tau, total tau; p-tau_181_*, *hyperphosphorylated tau; Aβ, beta-amyloid.**

### Covariate Effects

For the neurobehavioral factor scores, older age was associated with lower visual memory (β = −0.36, *p* = 0.01) and behavioral/mood (β = −0.38, *p* = 0.01) factor scores. Higher WRAT-4 scores contributed to the prediction of higher psychomotor speed and executive function (β = 0.37, *p* < 0.01) and verbal memory (β = 0.31, *p* = 0.03) factor scores. *APOE* ε4 carriers had lower visual memory scores (mean difference = 0.64, *p* = 0.01) compared to non-carriers. Higher BMI corresponded to lower psychomotor speed and executive function factor scores (β = −0.32, *p* = 0.01).

For the MRI-derived volumetric measures, older age was associated with lower left (β = −0.28, *p* = 0.03) and right (β = −0.29, *p* = 0.002) hippocampal volume, as well as greater volume of WM hypointensities (β = 0.30, *p* = 0.02). Higher systolic blood pressure was also associated with lower right hippocampal volume (β = −0.26, *p* = 0.02). Estimated ICV correlated with GMV (β = 0.49, *p* < 0.01) and WMV (β = 0.38, *p* < 0.01).

For the CSF analytes, the only association was between older age and higher CSF t-tau concentrations (β = 0.29, *p* = 0.03).

There were no statistically significant effects for years of education on the outcomes.

### Race × CHII Effects

**FIGURE 1:**
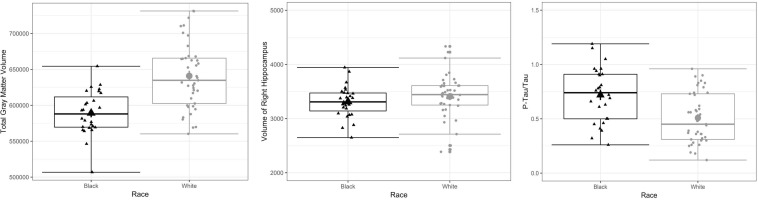
Box Plots on Differences Between Black and White Former NFL Players on Total Gray Matter Volume, Right Hippocampal Volume, and CSF p-tau_181_/t-tau. The mid-point line in the box represents the median, the interquartile range box represents the middle 50%, and the whiskers represent the bottom and top 25% of data values. The box plots show unadjusted associations. The multivariate generalized linear models showed a statistically significant main effect for race on these outcomes (FDR-adjusted *p*-value less than 0.05) after controlling for age, years of education, WRAT-4 scores, body mass index (BMI), systolic blood pressure, history of diabetes, and *APOE* status (ε4 carriers vs. non-carriers). Estimated intracranial volume (ICV) was also included as a covariate for total gray matter volume and right hippocampal volume.

**FIGURE 2:**
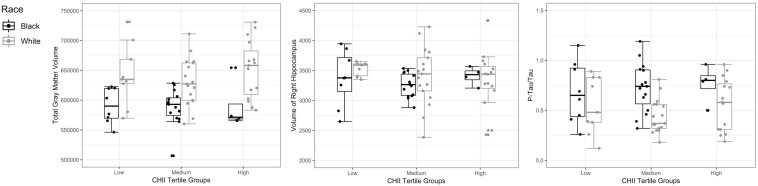
Race by CHII Interaction Effects. Multivariate generalized linear models showed a significant Race × CHII interaction for the plotted outcomes. The x-axis are CHII score tertiles. The mid-point line in the box represents the median, the interquartile range box represents the middle 50%, and the whiskers represent the bottom and top 25% of data values. There was a statistically significant association between the Race by CHII interaction term with the respective outcomes after controlling for age, years of education, WRAT-4 scores, body mass index (BMI), systolic blood pressure, history of diabetes, and *APOE* status (ε4 carriers vs. non-carriers). Estimated intracranial volume was also included as a covariate for total gray matter volume and right hippocampal volume. Note that race group differences at each CHII group were not examined due to insufficient sample size.

In terms of significant differences as a function of racial identity, compared to White former NFL players, Black participants had lower GMV (mean adjusted difference = 45649.00, *p* = 0.001), lower right hippocampal volume (mean adjusted difference = 271.96, *p* = 0.02), and higher p-tau_181_/t-tau ratio (mean adjusted difference = −0.25, *p* = 0.01). See Table 4, as well as Figure 1 for box plots of group differences. There was not a statistically significant association between the CHII and these outcomes (i.e., GMV, right hippocampal volume, p-tau_181_/t-tau ratio) in the entire sample. As shown in Table 5, there was a statistically significant Race x CHII interaction for the same outcomes for which there were race group main effects: GMV (*p* = 0.001), right hippocampal volume (*p* = 0.04), and p-tau_181_/t-tau ratio (*p* = 0.004). Figure 2 plots the race group differences on these outcomes based on CHII severity scores (i.e., low, medium, and high). There were no other statistically significant interaction effects. As shown in Figure 2, race group differences became magnified among those with higher CHII scores for GMV and CSF p-tau_181_/t-tau ratio; although, there was not such a linear effect for right hippocampal volume.

**TABLE 4:** Summary of multivariate generalized linear models comparing black and white symptomatic former NFL players on neurobehavioral measures, MRI-derived volumetric measures, and CSF levels of beta-amyloid, total tau, and P-tau.

	**Beta**	**Standard**	***T*-value**	***P*-value**
	**(unstandardized)**	**error**		**(FDR-adjusted)**
**Neurobehavioral function**				
Psychomotor speed/Executive function	0.45	0.21	2.14	0.15
Verbal memory	0.28	0.26	1.07	0.58
Visual memory	0.001	0.27	0.00	0.99
Behavior/Mood	0.13	0.25	0.51	0.82
**Volumetric measures**				
Total gray matter volume	45649.00	11000.00	4.15	0.001
Total cortical white matter volume	29686.00	12969.00	2.29	0.05
Total subcortical gray matter volume	190.45	1237.81	0.15	0.88
Right hippocampal volume	271.96	96.42	2.82	0.02
Left hippocampal volume	146.65	107.11	1.37	0.21
Volume of white matter hypointensities	–1204.35	582.43	–2.07	0.06
**CSF analytes**				
t-tau	4.73	3.67	1.29	0.20
p-tau_181_	–4.83	2.97	–1.63	0.18
Aβ_1__–__42_	33.32	23.36	1.43	0.20
p-tau_181_/t-tau	–0.25	0.07	–3.40	0.01
p-tau_181_/Aβ_1__–__42_	–0.02	0.01	–2.00	0.05

**Three separate multivariate Generalized Linear Models were performed to examine the racial identity differences on neurobehavioral variables, MRI-derived volumetric measures, and CSF analytes. All analyses controlled for age, years of education, WRAT-4 scores, body mass index (BMI), systolic blood pressure, history of diabetes, APOE status (ε4 carriers vs. non-carriers), and the cumulative head impact index (CHII). The MRI volumetric models also controlled for estimated intracranial volume. For neurobehavioral measures, lower scores are worse for all measures except for behavior/mood where higher scores are worse. Race is coded as 1 = Black and 0 = White and the beta estimates shown are White – Black. Abbreviations: FDR, false discovery rate; t-tau, total tau; p-tau, phosphorylated tau; Aβ, beta-amyloid.**

**TABLE 5:** Summary of multivariate generalized linear models examining the interaction between race and CHII on neurobehavioral measures, MRI-derived volumetric measures, and CSF levels of beta-amyloid, total tau, and P-tau.

	**Beta**	**Standard**	***T*-value**	***P*-value**
	**(unstandardized)**	**error**		**(FDR-adjusted)**
**Neurobehavioral function**				
Psychomotor speed/executive function	0.02	0.01	1.56	0.49
Verbal memory	0.01	0.01	1.05	0.59
Visual memory	–0.001	0.01	–0.10	0.92
Behavior/Mood	0.01	0.01	0.77	0.59
**Volumetric measures**				
Total gray matter volume	2206.29	543.07	4.06	0.001
Total cortical white matter volume	1137.01	647.51	1.76	0.17
Total subcortical gray matter volume	31.81	60.73	0.52	0.60
Right hippocampal volume	12.07	4.79	2.52	0.04
Left hippocampal volume	4.95	5.31	0.93	0.43
Volume of white matter hypointensities	–29.06	29.34	–0.99	0.43
**CSF analytes**				
t-tau	0.29	0.18	1.61	0.15
p-tau_181_	–0.21	0.14	–1.46	0.15
Aβ_1__–__42_	1.69	1.13	1.49	0.15
p-tau_181_/t-tau	–0.01	0.004	–3.50	0.004
p-tau_181_/Aβ_1__–__42_	–0.0008	0.0004	–1.90	0.15

**Three separate multivariate generalized linear models were performed to examine the effect of the Race x CHII interaction term on neurobehavioral variables, MRI-derived volumetric measures, and CSF analytes. All analyses controlled for age, years of education, WRAT-4 scores, body mass index (BMI), systolic blood pressure, history of diabetes, and APOE status (ε4 carriers vs. non-carriers). The MRI volumetric models also controlled for estimated intracranial volume. For neurobehavioral measures, lower scores are worse for all measures except for behavior/mood where higher scores are worse. Race is coded as 1 = Black and 0 = White. Abbreviations: FDR, false discovery rate; t-tau, total tau; p-tau, phosphorylated tau; Aβ, beta-amyloid.**

## Discussion

In this sample of symptomatic former NFL players, higher levels of exposure to RHI (as defined by the CHII) and Black racial identity had an interactive effect on GMV, right hippocampal volume, and CSF p-tau_181_/t-tau ratio concentrations. Although there was not a statistically significant association between CHII scores with these outcomes, Black former NFL players had lower GMV, lower right hippocampal volume, and higher CSF p-tau_181_/t-tau ratio concentrations compared to White former NFL players. There were no statistically significant main or interaction effects on the neurobehavioral factor scores. All effects were independent of age, years of education, WRAT-4 scores, CVD risk factors, *APOE*ε4, and eICV (for MRI models). Further research is needed to clarify the nuanced role of racial neurological disparities in this setting by examining the role of socioeconomic, psychosocial, environmental, and genetic variables that were not measured in this study.

Black former NFL players who had high CHII scores also had higher (on average) p-tau_181_/t-tau concentrations. P-tau_181_ is a biomarker of NFTs and the p-tau_181_/t-tau ratio has been shown to be a sensitive biomarker for the detection of tauopathies that are similar to CTE (e.g., FTLD) (Meeter et al., 2018). It is interesting that there was a specific Race x CHII effect for CSF p-tau_181_/t-tau but not t-tau or p-tau_181_. This could be indicative of an early neurodegenerative disease process among the subset of Black participants who have higher levels of exposure to RHI. Individuals who identify as Black have been shown to have decreased resistance to neurodegenerative disease pathology (Graff-Radford et al., 2016; Filshtein et al., 2019). Exposure to RHI from contact sports has also been associated with CTE and other neurodegenerative diseases (McKee et al., 2013; Bieniek et al., 2015; McKee et al., 2016; Alosco et al., 2019a; Ling et al., 2017; Mez et al., 2017b; Adams et al., 2018; Tagge et al., 2018; Stern et al., 2019). Not everyone who is exposed to RHI will develop later-life neurological disorders. Previous fluid biomarker research by our team (Alosco et al., 2018c) and others (Asken et al., 2018b) emphasizes that other risk factors are likely at play, which may include those that occur more frequently in individuals with Black racial identity (e.g., CVD, low SES). In this study, the CHII only had an association with CSF p-tau_181_/t-tau ratio through its interaction with Black racial identity. Explanations for the lack of effects of the CHII on p-tau_181_ (and other CSF biomarkers) in the DETECT sample have been provided elsewhere (Alosco et al., 2018c). There was not a Race x CHII effect on Aβ_1__–__42_ or p-tau_181_/Aβ_1__–__42,_ nor were there race group differences for Aβ_1__–__42_. While such findings argue against an AD pathway, racial identity and RHI likely have independent and combined associations with mixed neurodegenerative disease processes that cannot be disentangled here. Inferences specifically regarding CTE cannot be made given it cannot yet be diagnosed during life and the utility and validity of *in vivo* fluid and imaging biomarkers in CTE remain unclear (Stern et al., 2019).

Although Black participants had lower GMV and right hippocampal volume compared to White participants, there was not a main effect of CHII on these outcomes and the magnitude and direction of the interaction between race and CHII was less clear compared to CSF p-tau_181_/t-tau, particularly for right hippocampal volume (see Figure 1). There were also no other Race x CHII interaction effects. Our pattern of findings could be a consequence of being statistically underpowered, particularly given that there were few Black participants who had high CHII scores. Alternatively, recruitment and eligibility methods for the DETECT study were not designed to examine issues pertaining to race. Our recruitment (e.g., postings and presentations to the NFL Players and Alumni Associations, social media postings through our academic center outlets) and eligibility (e.g., English-speaking only) methods may have resulted in enrollment of socioeconomically and culturally homogeneous participants. Participants were also recruited based on self-reported symptomatic status, which was done via informal assessment and not quantitated. It is unclear if there were racial group differences in recruitment based on symptoms. Helmet accelerometer data from the college level were also extrapolated and used for this professional sample, which may have underestimated the effects of RHI, particularly at the professional levels. Other RHI exposure variables are also not included in the CHII (e.g., severity, interval rest, impact location). There are possible conceptual explanations, including that any effects observed were subclinical; this is supported by the largely normal neuropsychological test performance in the sample. Previous research among collegiate athletes has also shown that the association between CNS pathology (based on serum biomarker concentrations) and clinical function may actually be mediated by racial identity (Asken et al., 2018a). Lastly, because all of the former NFL players had extreme levels of exposure to RHI, this common risk factor might attenuate any pre-existing racial group differences and Race by CHII interactions that might exist for some neurological markers.

We *a priori* selected covariates associated with race and neurological outcomes. Age made a significant contribution to the prediction of many of the outcomes. *APOE* status, WRAT-4 scores, and BMI were associated with aspects of cognitive function, and systolic blood pressure correlated with right hippocampal volume. Years of education was not associated with any of the outcomes. When studying elite American football players, years of education might not be an adequate marker of SES as most play 4 years of college football. This was exemplified by the restricted range and lack of race group differences in education years in this sample. As a result of the DETECT study not being designed to examine racial disparities, detailed assessments of socioeconomic, language, cultural, and psychosocial history were not performed. There are thus many unmeasured variables related to racial identity that were not accounted for, which limit the validity of our findings and result in an incomplete understanding of the observed neurological racial disparities. Lower socioeconomic status, chronic health conditions and decreased health literacy (Verney et al., 2019), worse early-life education quality (Sisco et al., 2015) and fewer years of education (Weuve et al., 2018), perceived discrimination (Zahodne et al., 2017), geographical location (Liu et al., 2015), and genetic variations other than *APOE*ε4 (Lee et al., 2007; Logue et al., 2014; Mez et al., 2017a; Yu et al., 2017a, b) all contribute to increased vulnerability to cognitive decline among Black participants (Zahodne et al., 2017). These variables may have increased salience in American football players and thus have important clinical implications (Asken et al., 2016, 2017; Allison et al., 2018). Nuanced approaches (Galea et al., 2010) that model the multilevel interactions among social, environmental, genetic, and biological variables will elucidate racial heterogeneity associated with brain aging (in all settings).

A common explanation for the association between Black racial identity and neurological disorders is the higher rates of CVD risk factors (e.g., hypertension, diabetes, obesity) (Newman et al., 2005; Jackson et al., 2013; Barnes and Bennett, 2014; Benjamin et al., 2019) and cerebrovascular disease in Black participants (Zahodne et al., 2015; Graff-Radford et al., 2016; Filshtein et al., 2019). CVD is prevalent in former NFL players (Rogers et al., 2017) where it is a leading cause of mortality (Lehman et al., 2012, 2016; Lincoln et al., 2018). In this sample, Black participants had a higher BMI and higher systolic blood pressure. Yet, we observed effects after controlling for key CVD risk factors and there were minimal associations between the CVD risk factors and the outcomes. This could be related to imprecise measurement of adiposity and vascular health and/or the low rates of CVD. Additionally, racial disparities in CVD have been related to neighborhood conditions, access to and quality of medical care, and lifestyle behaviors, such as poor diet (Chin et al., 2011; Howard et al., 2018). These are variables that need to be included in future research to obtain a holistic understanding of racial disparities as it relates to CVD and brain aging, in general, and to CTE and related disorders, in particular.

Continued active engagement of Black participants in CTE-related research is encouraged to facilitate the study of various psychosocial, lifestyle, and genetic risk factors. Appropriate representation depends on the targeted football population being studied (e.g., active vs. former; college vs. NFL), given the changes of the racial make-up across levels of play and over time. The proportion of older adult (ages 40–69) former Black NFL players in this sample (i.e., 40%) is consistent with the rates of Black former NFL players reported in the mortality cohort studies of NFL players who played between 1959 and 1988 (Lehman et al., 2012, 2016). However, ∼70% of active NFL players today are Black (Lapchick and Marfatia, 2017; Lincoln et al., 2018). There are challenges and barriers to the recruitment, enrollment, and retainment of Black participants in research (Shavers et al., 2000; Braunstein et al., 2008; Byrd et al., 2011; Barnes et al., 2012; Barnes and Bennett, 2014), some of which may have affected screening, selection, and retention of participants in this study. Progress has been made via methodological frameworks put forth by the National Institute on Aging’s Health Disparities Framework (Hill et al., 2015) and recommendations provided by others (Barnes et al., 2012; Jefferson et al., 2013; Sabir and Pillemer, 2014; Samus et al., 2015; Brewster et al., 2018). Multipronged recruitment approaches anchored in establishing and maintaining trust of racially and ethnically diverse communities are advocated.

The generalizability of the findings is limited to symptomatic former NFL players and may not extend to other former NFL players, the broader American football population, or the general community. A complex system models approach (Galea et al., 2010) has been recommended (Brewster et al., 2018) to simultaneously evaluate the different variables and pathways that interact with self-identified race to influence neurological outcomes. This approach is computationally intensive and more suited to large multiple-source epidemiological datasets. The small sample size of the Black and White subgroups in this study additionally limits the ability to obtain reliable path estimates using statistical techniques such as structural equation modeling. Longitudinal research among large samples of former American football players (across all levels of play) are needed to validate our findings, elucidate race-moderated pathways of neurodegeneration and cognitive impairment, examine racial differences in trajectories of neurological outcomes, and identify the biopsychosocial variables that might contribute to observed race group differences. There were missing data across the different outcome variables, resulting in a reduced sample size and generalizability. Although the results remained largely similarly when the outcomes were analyzed based on complete data for that specific outcome (as opposed to complete data across all outcomes), there was some loss of statistical significance after restricting the sample to those who had complete data across all measures. Therefore, lack of statistical power may have precluded the ability to detect all associations. Unmeasured factors associated with missingness may have also affecting the accuracy of the estimated effects.

## Conclusion

In this sample of symptomatic former NFL players, Black racial identity and RHI had an interactive effect on GMV, right hippocampal volume, and CSF p-tau_181_/t-tau concentration. Although there were no statistically significant associations between exposure to RHI and these outcomes, Black participants had lower GMV, lower right hippocampal volume, and higher CSF p-tau_181_/t-tau compared to White participants. Future investigations are needed to model the complex role(s) of social, economic, environmental, and genetic variables in the association among race, RHI, and neurological outcomes.

## Data Availability Statement

The dataset generated for this study is available on request to the corresponding author, as well as with the completion of a data use agreement.

## Ethics Statement

The studies involving human participants were reviewed and approved by all study protocols were approved by the Boston University Medical Center Institutional Review Board. The Partners Institutional Review Board approved all neuroimaging procedures. The patients/participants provided their written informed consent to participate in this study.

## Author Contributions

MA, YT, AC, JJ, MMa, BS, and RS contributed to the study design and conception. MA, YT, AC, JJ, JM, MMa, OH, ÉF, BM, JP, IK, KG, NM, CL, MMu, AL, MC, OP, SB, MS, and RS contributed to data acquisition, analysis, and interpretation of data. All authors have contributed to drafting and critically revising the manuscript. All authors have given final approval of the version to be published and agreed to be accountable for the work.

## Conflict of Interest

RS was a member of the Mackey-White Committee of the NFL Players Association. He is a paid consultant to Biogen (Cambridge, MA, United States) and Eli Lilly (Indianapolis, IN, United States). He receives royalties for published neuropsychological tests from Psychological Assessment Resources, Inc. (Lutz, FL, United States) and was a member of the Board of Directors of King-Devick Technologies (Chicago, IL, United States). MA has received honorarium as a Scientific Advisor for Corino Therapeutics, Inc. The remaining authors declare that the research was conducted in the absence of any commercial or financial relationships that could be construed as a potential conflict of interest.

## Footnotes

1 http://surfer.nmr.mgh.harvard.edu

2 http://www.slicer.org/
